# Engaging People with Lived Experience: Co‐Creating Solutions for Dementia Programs in Colombia

**DOI:** 10.1002/alz70860_097900

**Published:** 2025-12-23

**Authors:** Sandra Baez

**Affiliations:** ^1^ Universidad de los Andes, Bogotá, Colombia, Bogota, Bogota, Colombia; Global Brain Health Institute (GBHI), Trinity College Dublin, Dublin, Dublin, Ireland

## Abstract

Caregiving for people living with dementia in Latin America, particularly in Colombia, poses significant challenges due to socioeconomic inequality, fragile healthcare systems, and stigma. Informal caregiving, predominantly undertaken by women, results in heightened stress, depression, and physical health problems. Despite these challenges, patient and public involvement (PPI) approaches are underutilized in Colombia. PPI involves active collaboration with caregivers, at all stages of research, including refining research questions, designing protocols, co‐creating interventions, and disseminating findings

This presentation highlights preliminary findings from a project aimed at enhancing the wellbeing of women dementia caregivers from low socioeconomic backgrounds through a three‐step process. Step 1 involves identifying psychosocial factors like life satisfaction, psychological wellbeing, caregiver burden, depressive symptoms, and patient‐related factors. Step 2 focuses on co‐creating a tailored intervention with caregivers, experts, and policymakers, including psychoeducation, mindfulness practices, and guidance on caregiving responsibilities. Step 3 tests the intervention in a feasibility study with 24 participants to refine its components and assess its effectiveness. The intervention, co‐created with caregivers, experts, and policymakers, is anticipated to include psychoeducation, mindfulness practices, guidance on managing caregiving responsibilities, and access to health resources. PPI has been integral to this project, with a panel of three caregivers invited through connections with social leaders.

Preliminary results highlight critical challenges, including severe caregiver burden, lack of financial resources and support, and barriers to participation, such as difficulty leaving home. The PPI panel emphasized the project's relevance and valued its attention to contextual needs. These caregivers contributed to shaping the study design, refining participant materials, and suggesting effective recruitment strategies. Insights from this project that are relevant to other countries and contexts will be illustrated. This ongoing initiative underscores the importance of culturally sensitive, gender‐focused interventions and demonstrates how inclusive approaches can help address caregiver needs by informing dementia research.

